# Supplementary material for: Study of the psychometric properties of the HLS-EU-12 questionnaire in rural Bangladesh
Source: PLoS One. 2025 May 12;20(5):e0323608. doi: 10.1371/journal.pone.0323608 (PMC12068572; doi:10.1371/journal.pone.0323608)
Supplement: Supplement 1 — HLS questionnaire Bengali and English versions.pdf (Pdf) [file pone.0323608.s002.pdf]

Section 2: Health literacy questionnaire (HLS-EU-Q12 from HLS-EU-Q47 version

On a scale from very difficult to very easy, how difficult would you say it is to:

		Very difficult	Fairly difficult	Fairly easy	Very easy
Q 1	…find information on treatments of illnesses that concern you?				
Q 2	…understand the leaflets that come with your medicine?				
Q 3	…judge the advantages and disadvantages of different treatment options?				
Q 4	…call an ambulance in an emergency?				
Q 5	…find information on how to manage mental health problems like stress or depression?				
Q 6	…understand why you need health screenings (such as breast exam, blood sugar test, blood pressure)?				
Q 7	…judge which vaccinations you may need?				
Q 8	…decide how you can protect yourself from illness based on advice from family and friends?				
Q 9	…find out about activities (such as meditation, exercise, walking, Pilates etc) that are good for your mental well-being?				
Q 10	…understand information in the media (such as Internet, newspaper, magazines) on how to get healthier?				
Q 11	…judge which everyday behavior (such as drinking and eating habits, exercise etc.) is related to your health?				
Q 12	… join a sports club or exercise class if you want to?				

সেকশন ২: স্বাস্থ্য সাক্ষরতার প্রশ্নাবলী

	খুব অসুবিধা	অসুবিধা	মোটামুটি সহজ	খুব সহজ
প্রশ্ন 1… অসুস্থতার চিকিত্সা সম্পর্কিত তথ্য সন্ধান করা আপনার জন্য অসুবিধা বা সুবিধা কি রকম হয় বলুন?				
প্রশ্ন 2… আপনার ওষুধের সাথে আসা লিফলেটগুলি বুঝতে কি অসুবিধা বা সুবিধা কি রকম হয় বলুন?				
প্রশ্ন 3… বিভিন্ন চিকিত্সার বিকল্পগুলির সুবিধা এবং অসুবিধাগুলি বিচার করতে কিভাবে পারেন?				
প্রশ্ন 4 … জরুরী পরিস্থিতিতে একটি অ্যাম্বুলেন্স পাওয়া কতটা সহজ?				
প্রশ্ন 5 … মানসিক স্বাস্থ্য সমস্যাগুলি যেমন মানসিক চাপ বা হতাশাগুলি পরিচালনা করার জন্য তথ্য জানা আপনার জন্য অসুবিধা বা সুবিধা কি রকম হয় বলুন?				
প্রশ্ন 6 … আপনার স্বাস্থ্য পরীক্ষা কেন প্রয়োজন তা বুঝতে (যেমন স্তন পরীক্ষা, রক্তে শর্করার পরীক্ষা, রক্তচাপ) অসুবিধা বা সুবিধা কি রকম হয় বলুন?				
প্রশ্ন 7 … আপনার কোন ভ্যাকসিনের প্রয়োজন হতে পারে তা বিচার করা অসুবিধা বা সুবিধা কি রকম হয় বলুন?				

প্রশ্ন ৪ ... আপনি পরিবার এবং বন্ধুদের পরামর্শের ভিত্তিতে অসুস্থতা থেকে নিজেকে রক্ষা করতে পারাটা অসুবিধা বা সুবিধা কি রকম হয় বলুন?				
প্রশ্ন 9 ... আপনার মানসিক সুস্থতার জন্য ভাল এমন ক্রিয়াকলাপগুলি (যেমন ধ্যান, অনুশীলন, হাঁটাচলা ইত্যাদি) সম্পর্কে তথ্য প্রাপ্তি কি অসুবিধা বা সুবিধা?				
প্রশ্ন 10... মিডিয়াতে (যেমন ইন্টারনেট, সংবাদপত্র, ম্যাগাজিনের মতো) কীভাবে স্বাস্থ্যকর হতে পারে সে সম্পর্কে তথ্য বুজতে অসুবিধা বা সুবিধা কি রকম হয় বলুন?				
প্রশ্ন 11... আপনার প্রতিদিনের স্বাস্থ্যের সাথে সম্পর্কিত আচরণ (যেমন পানীয় এবং খাওয়ার অভ্যাস, অনুশীলন ইত্যাদি) বিচার করতে পারা কি অসুবিধা বা সুবিধা কি রকম হয় বলুন?				
প্রশ্ন 12... যদি একটি স্পোর্টস ক্লাবে যোগ দিতে বা অনুশীলন ক্লাস আপনি যেতে চান, এ সুবিধা গুলো কি রকম? অসুবিধা বা সুবিধা কি রকম হয় বলুন।				
