# Supplementary material for: Study of the psychometric properties of the HLS-EU-12 questionnaire in rural Bangladesh
Source: PLoS One. 2025 May 12;20(5):e0323608. doi: 10.1371/journal.pone.0323608 (PMC12068572; doi:10.1371/journal.pone.0323608)
Supplement: Attachment — Submitted filename: Review.docx [file pone.0323608.s003.docx]

Dear editor and authors,

Thank you for the opportunity to review this article. The Rasch analysis of this article presents arguments supporting that the health literacy scale under investigation has good measurement properties. However, methods and results need to be revised thoroughly, main critical points are an elusive method section and a somewhat unstructured reporting of the results. The authors have to be more explicit in describing how compliance to measurement assumptions was determined, i.e. which tests, which cut-offs and why. Also, it is not totally clear how the analysis of unidimensionality was done, and aspects of targeting are spread across the method and result sections. I would suggest to follow a clearer structure, in describing the model fit, reliability and targeting, the fit of the items and the ordering of their response options, the local item dependencies and dimensionality, as well as the differential item functioning.

The categorization of the interval scaled score may need more justification. Why are scores below 50 seen as inadequate, the smaller range from 50-65 as problematic, and the 66-84 range as sufficient? How did the authors come up with these values, it seems to be based on literature – reference is missing – a sentence on the approach could be added (is it distribution based?).

Would density curves for the scores, by gender or age group etc. not be an option with higher validity instead of barcharts for the categories?

Given that data fits the Rasch model, I would recommend considering a linear regression analysis with the interval scaled scores instead of a logistic regression where the scores have been grouped in two categories.

The English needs to be revised by a native speaker.

The authors provide good and interesting summary tables. The labels need to be adjusted and text generally controlled for typos. Correction for repeated measures have been applied whenever needed. Figure 2 is not really needed.

Here a few more comments and suggestions for correction regarding the manuscript (in order of appearance in the text).

"and the tool validated unidimensionality" -> the analysis supported unidimensionality of the scale.

Introduction:

I would delete following information that do not relate anymore to the topic of assessment of health literacy and only reports some instruments that were evaluated using the Rasch model:

Suggest to remove: "Uddin et al. [21-24] used Rasch analysis to validate the Kessler 10-item (K10) questionnaire and the World Health Organization Quality of Life (WHOQoL) 26- item (WHOQoL 26) questionnaire. They proposed a seven-item tool (K7) and 18-item (WHOQoL 18) tool, respectively in rural Bangladesh."

Page 7: 2 | MATERIALS AND ETHODS –> and METHODS

Page 8: CVD – please write it out.

Page 8: I agree, that for a 12-item tool, a sample of 300 may be sufficient. However, I am not sure if this formula is appropriate to estimate sample sizes in Rasch analysis. Why E = 0.08? Do you have a reference for using this formula in the context of Rasch analysis with polytomous items? (As I do not know, would this formula find the sample size required to conduct certain DIF analyses with higher certitude?).

Page 9: Regarding the recruitment, if the division consisting of an intervention and an control group is relevant for the scale validation, and as you mention it, I would also suggest either adding a DIF analysis for belonging to one or the other group or showing more characteristics of these groups (I am tempted to hypothesize, that the intervention group may have (acquired) higher health literacy, given their exposure to health problems and health interventions. I wonder, regarding the logistic regression, what percentage of the group with score < 50 are from the control group etc…) These two groups were not mentioned anymore later in the article.

Page 11: Suggest not to list the items in the text and rather refer to a table with the 12 items and response frequencies and percentages for each response option (maybe disaggregated by control and test group). The table can be shown in the results as part of the descriptive analysis. Meaning that the paragraph about the development of the Health Literacy tool could stop after the sentence "Finbraten et al.". Are there already published psychometrics regarding the two versions of the reported scales? these could be reported here instead.

2.8 Outcome Measures:

Scale Validation:

"fit residuals" is not a statistical test also Rasch models cannot recategorize items.

"The partial credit model *recognizes that each item has its rating scale structure and* is the

generalization of the dichotomous Rasch model." -> The partial credit model (PCM) is a generalization of the dichotomous Rasch model that recognizes that each item has its rating scale structure.

"Disordered thresholds usually occur when individuals find it challenging to classify between response categories, especially." [delete: for the lack of appropriate ability].

"The Rasch model requires evaluating some assumptions to ensure an instrument has Rasch properties" - > The Rasch model requires evaluating some assumptions to ensure an instrument has good measurement properties

property c) -> absence of differential item functioning

with regard to the overall fit instead of "non-significant p-value indicates that the items' hierarchical orders are stable across all underlying attribute levels."

rather write:

"A non-significant p-value indicates that the data is fitting the PCM."

The Item-person interaction statistics inform already about the targeting of the instrument, how well the items can assess the ability range of the participants. I suggest to rephrase it in a perspective of an analysis of the targeting of the instrument, where the mean difficulty and mean ability should be close to 0, (< 1 logit difference), and the SD should be within the acceptable range of +/- 2.5SD.

Page 13: "The assumption unidimensionality occurs…" -> In presence of unidimensionality all items measure one common attribute. In Rumm2030, the unidimensionality is tested with a principal component analysis of the residuals, where 1^st^ eigenvalues < 1.8 (or 2) support the unidimensionality of the scale. (see later comment also on the result section and reporting of the dimensionality test)

"The local independence [54] can be tested by calculating the correlation coefficient

among residuals, which is expected to be unrelated.---" -> "The local independence [54] can be tested by calculating the correlation coefficient among residuals. Correlation below 0.3 indicate that two items are locally independent.

(Please see also the comment regarding result section and choice of cut-off)

"Any presence of DIF violates the requirement of independence [55]." -> Any presence… of invariance [55].

"A high PSI value indicates high reliability, indicating that the scale can separate persons along the latent trait [57]." -> A high PSI value (> 0.8?) indicates high reliability, indicating that the scale can separate persons along the latent trait [57].

"In Rasch analysis, targeting is crucial in developing or validating a scale. It refers to how well the scale captures a person's estimates based on location. The item difficulty and a person's ability are assessed by graphically and statistically comparing the distribution of the item threshold map" - > all the information regarding the targeting should be reported grouped.

I miss a section on the item fit – that could come after the model fit.

Page 14 – end of paragraph: "according to the method of the HLS-EU" -> would need a reference here.

2.9 Statistical analysis

Page 15 -"Sociodemographic factors, including sex, age, and level of education, were presented in proportion using simple descriptive statistics." -> The descriptive statistics in Table .. show the sociodemographic characteristics of the sample, including frequencies and percentages by sex, age, and level of education.

Next sentence: of these factors or of the sociodemographic factors

To some extent, I find it a pity that the authors, after the Rasch analysis and reporting good measurement properties of the scale, did not chose to apply a linear regression with the health-literacy score coded from 0-100, and instead opt for an aggregation first into 4 ordered categories (and then into two). By doing so, it was assumed that the categorical steps are homogeneous within groups. With lack of homogeneity within groups there is a risk to lose power and at worst to have an inaccurate estimation. I would want to know more on the score distributions (mean scores and variance), person parameter distribution and measurement error within these categories.

I am not sure why the authors prefer a logistic regression, after reporting a categorization into 4 (and not a multinomial regression). However, in any case, I would rather support the use of a linear model, with the 0-100 interval scaled scores. I am also fine, with the logistic regression, but would expect than a point in the discussion regarding potential limitations by doing so.

3.1 Participant's profile

is 80% of the sample unemployed or retired?

Psychometric properties of the HLS-EU-12

Page 16 - The PSI value for the 11 items, 0.756 with the same four response categories, did not show any significant improvements, indicating that removing item 12 has no prominent contribution in separating persons.

I do not agree, removing item 12, slightly increased the PSI, indicating that removing item 12 had no negative effect on the scale, to the contrary. Please rephrase accordingly.

The Unidimensionality… the method section did not describe how this analysis will be done and what cut-offs are going to be used. Please provide more information on your strategy. You applied the t-test, and lower bound being < 5% would support undimensionality, here also different approaches are possible, for example comparing the items loading positively and negatively on the first PCA component, or compare the items against items with misfit or negative correlations. How did you perform this analysis – if the PCA loadings were guiding the grouping of items, you could also report 1^st^ eigenvalue (or 1^st^ eig. < 1.8 also support unidimensionality).

Page 17 – What is meant by? "However, merging item 1 with 3 or 3 with 7 and for the remaining pairs when correlation coefficients were marginally higher than 0.30… ".

3.3 Health literacy and associated factors

"the female was associated", - > that does not sound appropriate. The text should be revised by a native speaker.

"one poor female housewife versus one middle – class male government employee" … Could you maybe here aggregate more and say that living in poverty, being female etc was more prevalent in the lower 5% while in the upper 5% (or also10% ) being male and employed, of younter age idk …etc was more likely to be associated with a higher health literacy score?

4 Discussion

"including a few correlation coefficients greater than 0.30, and item 12's fit residual

was significant." – including a few local item dependencies and misfit in the item assessing …

I am unsure if a PSI of 0.75 is still good – I would add the information in the method with references, usually 0.9 is excellent, 0.8 is good 0.7 is sufficient – however this may depend.

"There are, so far, no well-documented recommended critical values to conclude that indicate local dependency, and 0.30 is an arbitrary cutoff only [62-65], but a value of 0.30 has been used more often." Christensen & al recommend using a cut-off that adjusts for test length which consists in cut-off = mean residual correlation + 0.2. A cut-off of 0.3 is very relaxed, but I agree that it is commonly used.

"No DIF effect satisfies one of the underlying assumptions of Rasch models: that the estimated item parameters should be stable for different populations, such as males or females. " - > the absence of DIF supports that item parameter are stable across gender and age groups.

"The current study’s reliability was not excellent, possibly due to the targeting not being at the highest level." This final statement would contradict what is previously said regarding the PSI.

The discussion is very lengthy, and some elements of discussion would fit better in the introduction, or were already mentioned in the introduction (eg. Switzerland Taiwan example).

The authors could maybe emphasize why health literacy is important, the need for such a tool, how such a tool allows maybe to detect vulnerable groups, or specific type of info that causes difficulty. Maybe add also examples where interventions to improve the health literacy had a positive effect.
