# Supplementary material for: Study of the psychometric properties of the HLS-EU-12 questionnaire in rural Bangladesh
Source: PLoS One. 2025 May 12;20(5):e0323608. doi: 10.1371/journal.pone.0323608 (PMC12068572; doi:10.1371/journal.pone.0323608)
Supplement: Attachment — Submitted filename: PONE-D-24-39234_revision letter R1_Islam.docx [file pone.0323608.s005.docx]

Title: Study of the psychometric properties of the HLS-EU-12 questionnaire and its use in examining health literacy and the associated sociodemographic factors in rural Bangladesh

Dear Editor,

Thank you for your extra ordinary feedback. Following your feedback, I would consider the manuscript has reached to an upper level. Thank you again.

Please see my responses in BLUE. The feedback marked in RED related to sociodemographic factors and health literacy is not addressed.

Best regards

Fakir M Amirul Islam

A/Professor, School of Health Science

Swinburne University of Technology.

Editorial comments:

- A rebuttal letter that responds to each point raised by the academic editor and reviewer(s). You should upload this letter as a separate file labeled 'Response to Reviewers'.

Response: The manuscript is now formatted according to PLOS One guidelines

- A marked-up copy of your manuscript that highlights changes made to the original version. You should upload this as a separate file labeled 'Revised Manuscript with Track Changes'.

Response: It is done.

- An unmarked version of your revised paper without tracked changes. You should upload this as a separate file labeled 'Manuscript'.

Response: It is done.

If you would like to make changes to your financial disclosure, please include your updated statement in your cover letter. Guidelines for resubmitting your figure files are available below the reviewer comments at the end of this letter.

Response: I have followed the guideline.

If applicable, we recommend that you deposit your laboratory protocols in protocols.io to enhance the reproducibility of your results. Protocols.io assigns your protocol its own identifier (DOI) so that it can be cited independently in the future. For instructions see: [https://journals.plos.org/plosone/s/submission-guidelines#loc-laboratory-protocols](https://track.editorialmanager.com/CL0/https:%2F%2Fjournals.plos.org%2Fplosone%2Fs%2Fsubmission-guidelines%23loc-laboratory-protocols/1/010f01942b67e5a5-12eb4139-1705-43ae-a2e9-24a9e5343e4b-000000/-Muma0DRWyqwJTQQcreaEwG6AgFeToNvmH6lpVo7o0Q=192). Additionally, PLOS ONE offers an option for publishing peer-reviewed Lab Protocol articles, which describe protocols hosted on protocols.io. Read more information on sharing protocols at [https://plos.org/protocols?utm_medium=editorial-email&utm_source=authorletters&utm_campaign=protocols](https://track.editorialmanager.com/CL0/https:%2F%2Fplos.org%2Fprotocols%3Futm_medium=editorial-email%26utm_source=authorletters%26utm_campaign=protocols/1/010f01942b67e5a5-12eb4139-1705-43ae-a2e9-24a9e5343e4b-000000/CkJGDpE0NE4ulqGpcw3Z47UW37fiLFyERX4AI5WA1og=192).

Response: The protocol was published. The title and doi are given below.

doi: 10.1186/s13063-021-05379-2.

**Title: Lowering blood pressure by changing lifestyle through a motivational education program: a cluster randomized controlled trial study protocol**

Journal Requirements:

1. When submitting your revision, we need you to address these additional requirements.

   Please ensure that your manuscript meets PLOS ONE's style requirements, including those for file naming. The PLOS ONE style templates can be found at
   [https://journals.plos.org/plosone/s/file?id=wjVg/PLOSOne_formatting_sample_main_body.pdf](https://track.editorialmanager.com/CL0/https:%2F%2Fjournals.plos.org%2Fplosone%2Fs%2Ffile%3Fid=wjVg%2FPLOSOne_formatting_sample_main_body.pdf/1/010f01942b67e5a5-12eb4139-1705-43ae-a2e9-24a9e5343e4b-000000/rbqQXo3GwLJUmAwCyP95riSrJV1hKluLTdr6CRDzFvA=192) and
   [https://journals.plos.org/plosone/s/file?id=ba62/PLOSOne_formatting_sample_title_authors_affiliations.pdf](https://track.editorialmanager.com/CL0/https:%2F%2Fjournals.plos.org%2Fplosone%2Fs%2Ffile%3Fid=ba62%2FPLOSOne_formatting_sample_title_authors_affiliations.pdf/1/010f01942b67e5a5-12eb4139-1705-43ae-a2e9-24a9e5343e4b-000000/X0NREgAC9LaFx6EQTEQ4UC4e7GspvDzOkvlybTTwkB0=192)
   Response: I have formatted according to the Journal’s requirements.

2. Please ensure you have included the registration number for the clinical trial referenced in the manuscript.

Response: **Trial registration:** ClinicalTrials.gov [NCT04505150](http://clinicaltrials.gov/show/NCT04505150) . Registered on 7 August 2020.

2. When completing the data availability statement of the submission form, you indicated that you will make your data available on acceptance. We strongly recommend all authors decide on a data sharing plan before acceptance, as the process can be lengthy and hold up publication timelines. Please note that, though access restrictions are acceptable now, your entire data will need to be made freely accessible if your manuscript is accepted for publication. This policy applies to all data except where public deposition would breach compliance with the protocol approved by your research ethics board. If you are unable to adhere to our open data policy, please kindly revise your statement to explain your reasoning and we will seek the editor's input on an exemption. Please be assured that, once you have provided your new statement, the assessment of your exemption will not hold up the peer review process.

Response: Data are now made available.

Reviewer 1

Thank you for the opportunity to review this article. The Rasch analysis of this article presents arguments supporting that the health literacy scale under investigation has good measurement properties. However, methods and results need to be revised thoroughly, main critical points are an elusive method section and a somewhat unstructured reporting of the results. The authors have to be more explicit in describing how compliance to measurement assumptions was determined, i.e. which tests, which cut-offs and why. Also, it is not totally clear how the analysis of unidimensionality was done, and aspects of targeting are spread across the method and result sections. I would suggest to follow a clearer structure, in describing the model fit, reliability and targeting, the fit of the items and the ordering of their response options, the local item dependencies and dimensionality, as well as the differential item functioning.

Response: I have followed a clear structure and systematically presented the methods and results sections.

Response: I have focused on the tool's psychometric properties. Therefore, any feedback related to health literacy factors is irrelevant. The comments highlighted in red are not addressed.

The categorization of the interval scaled score may need more justification. Why are scores below 50 seen as inadequate, the smaller range from 50-65 as problematic, and the 66-84 range as sufficient? How did the authors come up with these values, it seems to be based on literature – reference is missing – a sentence on the approach could be added (is it distribution based?).

Would density curves for the scores, by gender or age group etc. not be an option with higher validity instead of barcharts for the categories?

Given that data fits the Rasch model, I would recommend considering a linear regression analysis with the interval scaled scores instead of a logistic regression where the scores have been grouped in two categories.

The English needs to be revised by a native speaker.

The authors provide good and interesting summary tables. The labels need to be adjusted and text generally controlled for typos. Correction for repeated measures have been applied whenever needed. Figure 2 is not really needed.

Response: Thanks. The manuscript is now read by a native English speaker. I have tried to format the Tables with fewer decimal places to make them look better and clearer.

Figure 2 is removed.

Here a few more comments and suggestions for correction regarding the manuscript (in order of appearance in the text).

"and the tool validated unidimensionality" -> the analysis supported unidimensionality of the scale.

Response: It is now corrected.

Introduction:

I would delete following information that do not relate anymore to the topic of assessment of health literacy and only reports some instruments that were evaluated using the Rasch model:

Suggest to remove: "Uddin et al. [21-24] used Rasch analysis to validate the Kessler 10-item (K10) questionnaire and the World Health Organization Quality of Life (WHOQoL) 26- item (WHOQoL 26) questionnaire. They proposed a seven-item tool (K7) and 18-item (WHOQoL 18) tool, respectively in rural Bangladesh."

Response: I have removed it.

Page 7: 2 | MATERIALS AND ETHODS –> and METHODS

Response: Thanks. It was a silly mistake.

Page 8: CVD – please write it out.

Response: It is done.

Page 8: I agree, that for a 12-item tool, a sample of 300 may be sufficient. However, I am not sure if this formula is appropriate to estimate sample sizes in Rasch analysis. Why E = 0.08? Do you have a reference for using this formula in the context of Rasch analysis with polytomous items? (As I do not know, would this formula find the sample size required to conduct certain DIF analyses with higher certitude?).

Response: Thanks. The sample size 307 (154 females) was fixed. I tried to show that at least 8 unit difference between any factor levels would produce a significant p with 80% power. Here, 8 is arbitrary; more than 8-unit difference for a sample size 150 by 150 is expected to show a significant p value.

Page 9: Regarding the recruitment, if the division consisting of an intervention and an control group is relevant for the scale validation, and as you mention it, I would also suggest either adding a DIF analysis for belonging to one or the other group or showing more characteristics of these groups (I am tempted to hypothesize, that the intervention group may have (acquired) higher health literacy, given their exposure to health problems and health interventions. I wonder, regarding the logistic regression, what percentage of the group with score < 50 are from the control group etc…) These two groups were not mentioned anymore later in the article.

Response: Thanks. This is a very good point. However, the data are from the baseline study when both groups were similar, and no intervention was implemented. I have added this information to Table 1.

Page 11: Suggest not to list the items in the text and rather refer to a table with the 12 items and response frequencies and percentages for each response option (maybe disaggregated by control and test group). The table can be shown in the results as part of the descriptive analysis. Meaning that the paragraph about the development of the Health Literacy tool could stop after the sentence "Finbraten et al.". Are there already published psychometrics regarding the two versions of the reported scales? these could be reported here instead.

Response: Now the title of the Table 1 is as follows:

Table 1. Responses to HLQ-EU questionnaire by the total participants and by study types, control vs. intervention group.

I have also included the following statement after "Finbraten et al.".

“The tool “HLS-EU-Q12” has not been validated using Rasch analysis in the Asian context, though Nguyen et al.  have provided evidence to support using Rasch analysis in health literacy instrument development [11]”.

2.8 Outcome Measures:

Scale Validation:

"fit residuals" is not a statistical test also Rasch models cannot recategorize items.

Response: I have used “parameters” instead of statistical test. I have changed the statement to

“Collapse or merge item categories, especially for disordered categories”.

"The partial credit model *recognizes that each item has its rating scale structure and* is the

generalization of the dichotomous Rasch model." -> The partial credit model (PCM) is a generalization of the dichotomous Rasch model that recognizes that each item has its rating scale structure.

Response: I have changed the statement as suggested.

"Disordered thresholds usually occur when individuals find it challenging to classify between response categories, especially." [delete: for the lack of appropriate ability].

Response: Done

"The Rasch model requires evaluating some assumptions to ensure an instrument has Rasch properties" - > The Rasch model requires evaluating some assumptions to ensure an instrument has good measurement properties

Response: Done

property c) -> absence of differential item functioning with regard to the overall fit instead of "non-significant p-value indicates that the items' hierarchical orders are stable across all underlying attribute levels."

rather write:

"A non-significant p-value indicates that the data is fitting the PCM."

The Item-person interaction statistics inform already about the targeting of the instrument, how well the items can assess the ability range of the participants. I suggest to rephrase it in a perspective of an analysis of the targeting of the instrument, where the mean difficulty and mean ability should be close to 0, (< 1 logit difference), and the SD should be within the acceptable range of +/- 2.5SD.

Response: I have made this change.

Page 13: "The assumption unidimensionality occurs…" -> In presence of unidimensionality all items measure one common attribute. In Rumm2030, the unidimensionality is tested with a principal component analysis of the residuals, where 1^st^ eigenvalues < 1.8 (or 2) support the unidimensionality of the scale. (see later comment also on the result section and reporting of the dimensionality test)

"The local independence [54] can be tested by calculating the correlation coefficient

among residuals, which is expected to be unrelated.---" -> "The local independence [54] can be tested by calculating the correlation coefficient among residuals. Correlation below 0.3 indicate that two items are locally independent.

(Please see also the comment regarding result section and choice of cut-off)

"Any presence of DIF violates the requirement of independence [55]." -> Any presence… of invariance [55].

"A high PSI value indicates high reliability, indicating that the scale can separate persons along the latent trait [57]." -> A high PSI value (> 0.8?) indicates high reliability, indicating that the scale can separate persons along the latent trait [57].

"In Rasch analysis, targeting is crucial in developing or validating a scale. It refers to how well the scale captures a person's estimates based on location. The item difficulty and a person's ability are assessed by graphically and statistically comparing the distribution of the item threshold map" - > all the information regarding the targeting should be reported grouped.

I miss a section on the item fit – that could come after the model fit.

Response: Now it is explained in a different section.

Page 14 – end of paragraph: "according to the method of the HLS-EU" -> would need a reference here.

2.9 Statistical analysis

Page 15 -"Sociodemographic factors, including sex, age, and level of education, were presented in proportion using simple descriptive statistics." -> The descriptive statistics in Table .. show the sociodemographic characteristics of the sample, including frequencies and percentages by sex, age, and level of education.

Response: In the statistical analysis section, I did not want to report the “Table”. I have written the statistical analysis section as below.

Descriptive statistics was used to show the sociodemographic characteristics of the sample, including frequencies and percentages of sex, age, and level of education by study groups (control vs. intervention). Chi-square tests were used to detect any association of the study groups with individual health literacy items and associations of sociodemographic factors with a binary cut-off, difficult and not difficult of the health literacy items. After psychometric properties validation of the HLS-EU 12 tool using Rasch analysis and confirming the 12 items retained, a combined score from all items was calculated on a continuous scale of 0 to 100, and its distribution was checked. Given that the distribution of the combined score followed approximately a normal distribution, a generalized linear model was used to measure the association of sociodemographic factors with the combined score and presented as estimated means with 95% confidence intervals by adjusting for covariates age, gender, study group, and education level.

To some extent, I find it a pity that the authors, after the Rasch analysis and reporting good measurement properties of the scale, did not chose to apply a linear regression with the health-literacy score coded from 0-100, and instead opt for an aggregation first into 4 ordered categories (and then into two). By doing so, it was assumed that the categorical steps are homogeneous within groups. With lack of homogeneity within groups there is a risk to lose power and at worst to have an inaccurate estimation. I would want to know more on the score distributions (mean scores and variance), person parameter distribution and measurement error within these categories.

I am not sure why the authors prefer a logistic regression, after reporting a categorization into 4 (and not a multinomial regression). However, in any case, I would rather support the use of a linear model, with the 0-100 interval scaled scores. I am also fine, with the logistic regression, but would expect than a point in the discussion regarding potential limitations by doing so.

Response: I have used the Generalized Linear model. The statistical analysis section and Table 6 are updated.

3.1 Participant's profile

is 80% of the sample unemployed or retired?

Response: Thanks. Sorry, I can’t find this information in the final submission.

The section is written as (Table 1 is new):

The participants' sociodemographic characteristics by study groups (control vs. intervention) show none of the variables was significant. For example, the proportion of females (47% in the control group vs. 53% in the intervention group, p=0.33) and no education (31% in the control group vs. 34% in the intervention group, p=0.52) were similar in the control and intervention group (Data are not shown in a Tabular format). Health literacy in individual items for the total sample and by study groups are shown in Table 1. Approximately 40% of the participants found it difficult in most of the items, with the item "…….judge which vaccinations you may need?" being the most difficult item (very difficult 23.8% and difficult 60.6%) and "…decide how you can protect yourself from illness based on advice from family and friends?" being the most easy item (fairy easy 66.1% and easy 24.8%). This baseline data showed no difference between the intervention and control groups.

Psychometric properties of the HLS-EU-12

Page 16 - The PSI value for the 11 items, 0.756 with the same four response categories, did not show any significant improvements, indicating that removing item 12 has no prominent contribution in separating persons.

I do not agree, removing item 12, slightly increased the PSI, indicating that removing item 12 had no negative effect on the scale, to the contrary. Please rephrase accordingly.

Response: I have rephrased it as:

The PSI value for the 11 items with the same four response categories, 0.756, a minor improvement from 0.746, indicates that removing item 12 had no negative effect on the scale.

The Unidimensionality… the method section did not describe how this analysis will be done and what cut-offs are going to be used. Please provide more information on your strategy. You applied the t-test, and lower bound being < 5% would support undimensionality, here also different approaches are possible, for example comparing the items loading positively and negatively on the first PCA component, or compare the items against items with misfit or negative correlations. How did you perform this analysis – if the PCA loadings were guiding the grouping of items, you could also report 1^st^ eigenvalue (or 1^st^ eig. < 1.8 also support unidimensionality). You applied the t-test, and lower bound being < 5% would support undimensionality, here also different approaches are possible, for example comparing the items loading positively and negatively on the first PCA component, or compare the items against items with misfit or negative correlations. How did you perform this analysis – if the PCA loadings were guiding the grouping of items, you could also report 1^st^ eigenvalue (or 1^st^ eig. < 1.8 also support unidimensionality).

Response: I have included the following information in the methods section with a reference.

One approach to test the unidimensionality is to divide the items based on the positive and negative factor loadings on the first PCA of residuals and estimate separate person measures based on the two item sets. Compare the two estimates on a person-by-person basis using *t*-tests to determine the number of cases differ significantly at the 0.05-level, or the lower bound of a binomial 95% confidence interval (CI) of the observed proportion overlaps 5%, then it is suggested a unidimensionality.

54.	Hagell P. Testing rating scale unidimensionality using the principal component analysis (PCA)/t-test protocol with the Rasch model: the primacy of theory over statistics. . Open J Stat 2014;4:456–65.

Page 17 – What is meant by? "However, merging item 1 with 3 or 3 with 7 and for the remaining pairs when correlation coefficients were marginally higher than 0.30… ".

3.3 Health literacy and associated factors

"the female was associated", - > that does not sound appropriate. The text should be revised by a native speaker.

"one poor female housewife versus one middle – class male government employee" … Could you maybe here aggregate more and say that living in poverty, being female etc was more prevalent in the lower 5% while in the upper 5% (or also10% ) being male and employed, of younter age idk …etc was more likely to be associated with a higher health literacy score?

Response: I have included the following statement.

Of the 5% of participants who had 80% or above difficulty, all had either no education or primary education. 60% of them were female, and 100% were housewives. Of the other 5% who had 20% or below difficulty, 90% were males, had SSC or above education, and were employed.

4 Discussion

"including a few correlation coefficients greater than 0.30, and item 12's fit residual

was significant." – including a few local item dependencies and misfit in the item assessing …

I am unsure if a PSI of 0.75 is still good – I would add the information in the method with references, usually 0.9 is excellent, 0.8 is good 0.7 is sufficient – however this may depend.

"There are, so far, no well-documented recommended critical values to conclude that indicate local dependency, and 0.30 is an arbitrary cutoff only [62-65], but a value of 0.30 has been used more often." Christensen & al recommend using a cut-off that adjusts for test length which consists in cut-off = mean residual correlation + 0.2. A cut-off of 0.3 is very relaxed, but I agree that it is commonly used.

Response: I have included the statement below with the reference [59].

A PSI value above 0.75 indicate good reliability, and values above 0.9 indicate very good or excellent reliability of the scale [59].

"No DIF effect satisfies one of the underlying assumptions of Rasch models: that the estimated item parameters should be stable for different populations, such as males or females. " - > the absence of DIF supports that item parameter are stable across gender and age groups.

Response: I have made this change.

"The current study’s reliability was not excellent, possibly due to the targeting not being at the highest level." This final statement would contradict what is previously said regarding the PSI.

Response: Thanks. I have claimed that the reliability was good, with a PSI value of 0.75. It needs to be 0.90 or more to be excellent, which is not. Therefore, I would consider that the statement was made based on the PSI value.

The discussion is very lengthy, and some elements of discussion would fit better in the introduction, or were already mentioned in the introduction (eg. Switzerland Taiwan example).

The authors could maybe emphasize why health literacy is important, the need for such a tool, how such a tool allows maybe to detect vulnerable groups, or specific type of info that causes difficulty. Maybe add also examples where interventions to improve the health literacy had a positive effect.

Response:

I have shortened the Introduction and discussion sections and updated them with the following information to show the importance of studying HL in Bangladesh. The introduction and discussion related to factors associated with HL are removed.

The study of health literacy in Bangladesh is minimal. Health service delivery is complex and involves a mix of public, private, and informally trained healthcare providers.  Bangladesh's education system is not equipped with the necessary skills to help people effectively interact with modern health systems and information to improve their health [32]. Studies suggest that specific interventions improve HL on health outcomes, including knowledge, self-efficacy, adherence, and quality of life [33].

Reviewer 2

1) Reviewer #2: Psychometric Study Review – Feedback and Suggestions

   Item Content Adjustment Post-Translation:
   The psychometric study seems well-conducted; however, I am surprised that no content adjustments were made to the items during translation. The HLS-Q12 was developed in a European population, which is culturally quite different from the Bangladeshi context. Could the authors provide justification for how the item content is well-suited to the Bangladeshi population, perhaps through expert opinions on health literacy in Bangladesh?

Response: I have presented the analyses and results more thoroughly.

The translated version of the questionnaire has now been submitted as a supplement.

Page 12:
The sentence:
“The HLS-EU-Q12 ranged from 19 to 44 with a mean of 30.3 (standard deviation (SD)=4.1) and a median of 30.0 from the range of 12 to 48 for 12 items with four categories.”
Is unclear in the context of Rasch model validation. Are we discussing "sum-scores," "standardized sum-scores," or latent traits here?

Response: Yes, it is not very clear. I have removed it.

Page 14:
"The combined score ranged from zero (very difficult in all items) to 100 (very easy to all items), categorized into two with scores 0-50, defined as ‘limited’, and 50 and above, defined as above average health literacy. The combined score is categorized into four levels: a health literacy test score of equal or below 50% is taken as ‘inadequate’ health literacy, 51-65 as problematic, 66-83 as sufficient and greater than or equal to 84 as excellent health literacy according to the method of the HLS-EU."
How were these thresholds defined? Are these the same thresholds used in Europe, and are they relevant for a Bangladeshi population? Were these thresholds redefined based on local data?

Response: Now, I have only focused on psychometric properties.

Page 16:
"Further analysis by removing item 12 showed a good overall fit with the Rasch model, as indicated by an insignificant item-trait interaction (χ² = 60.35 df = 44, p=0.051) (data not shown).”
The statement "showed a good fit" seems an overinterpretation of a test on the verge of significance. It would be prudent to acknowledge the limitations of fit tests, which tend to retain the null hypothesis of good fit, whereas hypothesis tests are more useful for rejecting the null hypothesis. A p-value of 0.051 suggests a lack of power rather than solid evidence for model fit.

Response: Thanks. I agree that there is no solid evidence for model fit. I have used this as a limitation. I have used a two-decimal P value (0.05).

I have used the limitation as:

fourthly, although removing item 12 showed a marginally significant p-value (0.05), model fit can be recommended with caution.

Unidimensionality Testing:
"The Unidimensionality of HLS-EU-12 was tested using principal component analysis (PCA) (5.2%, 95% CI 2.8% to 7.7%), indicating the lower bound was less than 5% (Table 2 (last row) and Fig. 3), which supports the Unidimensionality of the HLS-EU-12 item."
This sentence is unclear and does not explain that PCA was applied to residuals. What does the 5.2% represent? What reference supports the claim that this result confirms the unidimensionality of the questionnaire?

Response: I have included the following statements in the methods section to make the statement clear. “The lower bound should be more than 5% to report a multidimentionality”.

One approach to test the unidimensionality is to divide the items based on the positive and negative factor loadings on the first PCA of residuals and estimate separate person measures based on the two item sets. Compare the two estimates on a person-by-person basis using *t*-tests to determine the number of cases differ significantly at the 0.05-level, or the lower bound of a binomial 95% confidence interval (CI) of the observed proportion overlaps 5%, then it is suggested a unidimensionality.

Differential Item Functioning (DIF):
Table 3 suggests potential DIF for items 10 (age) and 2, 8, 10, 11 (gender), which contradicts the statement, "However, none of the items showed significant DIFs for age and gender, indicating the scale works equally for males and females, as well as for adults and older adults."

Response: Thanks. You are correct.

Page 17:
"However, merging item 1 with 3 or 3 with 7 and for the remaining pairs when correlation coefficients were marginally higher than 0.30 did not improve the overall model, indicated by the chi-square value. Thus, all the items are retained."
This sentence is unclear and needs rewording.

Response: Now it is written as

However, combining item pairs with correlation coefficients at least 0.30, such as item 1 with 3 and item 3 with 7, did not improve the overall model. Thus, all the items are retained.

Item 12 and Model Fit:
What should be done with item 12, which does not exhibit good fit?
Considering all these issues, can we compare the measures with those conducted in European countries?

Response: The article below, related to European data, reported a PSI of 0.75, precisely the same as I found in the current study. However, in the European study, HLS-Q12 did not find any occurrences of misfitting items.

Item 12, “… join a sports club or exercise class if you want to?” had significant fit residual, which warranted a considerable variation in responding to the item.  The reason for the misfit is unknown, but the question is related to personal judgment about safety, low crime rate, traffic safety, recreational facilities, and convenient places for joining sports clubs or physical activity programs, which are barriers in Bangladesh, and this can contribute to the misfit [69]. Further in-depth investigation is needed to understand this issue better.  However, removing item 12 had a minor improvement in the PSI, suggesting that the item had no significant negative impact on the model.

Health Literacy Determinants Study:
The study on determinants of low health literacy seems out of place. It is not appropriate to use the same study to both validate a questionnaire (i.e., validate a measure) and explore determinants (i.e., use a measure that is assumed to already be validated). This raises concerns about using the same results for both validating the measure (known-group validity) and explaining differences in the measure.

Response: Thanks for raising a good point. I have focused only on the psychometric properties.

Page 18:
The rationale for reintroducing item 12, which was previously shown to be incompatible with the measurement model, is unclear. Did this require any adaptations or statistical adjustments?

Response: I have discussed this finding

Page 20:
"HLS-SF12 can be a valid tool for measuring health literacy in rural areas of Bangladesh."
I am not entirely convinced by this statement.

Response: I agree with the reviewer. I have included the following statement to the conclusion

However, further study is needed to apply the tool to different populations, including an in-depth investigation of item 12 to determine its inclusion or removal.

DIF and Age/Gender:
"The HLS-Q12 showed no differential item functioning for gender or age groups."
This sentence does not align with the study results.

Response: Item-specific fit residuals are not significant. Our primary interest is to check if the items fit the psychometric properties.  I am not sure if the answer is satisfactory!!

Discussion:
All discussions regarding the use of this study to explore the determinants of health literacy (good or bad) are irrelevant. The conclusion should focus on the properties of the measure itself.

Response: The study checks the psychometric properties only.

Conclusion:
"The tool's adherence to Rasch assumptions of local dependency, unidimensionality, and invariance, coupled with its lack of DIF on age and sex, are significant."
This sentence is unclear and needs to be reworded.

Response: I have written this as

“Although the tool adheres to Rasch's assumptions of local dependency, unidimensionality, and invariance, further study is needed to check its psychometric properties in different populations.”

General Conclusion:
The idea that the measure is well validated should be reworked in the conclusion and summary. These results are not impressive from a psychometric perspective and should not be overstated. At best, we have a questionnaire that is "not too bad" psychometrically.

Response: Thanks.

Supplementary Documents:
It is essential for future Bangladeshi users of this questionnaire to have access to the translated version of the questionnaire.

Response: The questionnaire is attached as supplement.

These points highlight areas where further clarification, refinement, or additional information is necessary to enhance the psychometric rigor of the study and ensure its applicability to the Bangladeshi context.
