# Supplementary material for: Study of the psychometric properties of the HLS-EU-12 questionnaire in rural Bangladesh
Source: PLoS One. 2025 May 12;20(5):e0323608. doi: 10.1371/journal.pone.0323608 (PMC12068572; doi:10.1371/journal.pone.0323608)
Supplement: Attachment — Submitted filename: Revision letter R1_Editorial comments.docx [file pone.0323608.s006.docx]

Title: Study of the psychometric properties of the HLS-EU-12 questionnaire and its use in examining health literacy and the associated sociodemographic factors in rural Bangladesh

Dear Editor,

Thank you for your extraordinary feedback. I would like to mention that, according to the reviewers’ comments and my assessment, I took your opinion to focus on the psychometric properties only. Therefore, the comments related to factors associated with health literacy are no longer relevant. I have addressed the reviewers’ comments, focusing only on psychometric properties.

The manuscript title is

Title: Study of the psychometric properties of the HLS-EU-12 questionnaire in rural Bangladesh.

Please see my responses in BLUE.

Best regards

Fakir M Amirul Islam

A/Professor, School of Health Science

Swinburne University of Technology.

Editorial comments:

- A rebuttal letter that responds to each point raised by the academic editor and reviewer(s). You should upload this letter as a separate file labeled 'Response to Reviewers'.

Response: The manuscript is now formatted according to PLOS One guidelines

- A marked-up copy of your manuscript that highlights changes made to the original version. You should upload this as a separate file labeled 'Revised Manuscript with Track Changes'.

Response: The file is now named “Revision Manuscript with track changes_R1”

- An unmarked version of your revised paper without tracked changes. You should upload this as a separate file labeled 'Manuscript'.

Response: It is now labeled “Manuscript R1”

If you would like to make changes to your financial disclosure, please include your updated statement in your cover letter. Guidelines for resubmitting your figure files are available below the reviewer comments at the end of this letter.

Response: I have followed the PLOS one guideline to format the figures.

If applicable, we recommend that you deposit your laboratory protocols in protocols.io to enhance the reproducibility of your results. Protocols.io assigns your protocol its own identifier (DOI) so that it can be cited independently in the future. For instructions see: [https://journals.plos.org/plosone/s/submission-guidelines#loc-laboratory-protocols](https://track.editorialmanager.com/CL0/https:%2F%2Fjournals.plos.org%2Fplosone%2Fs%2Fsubmission-guidelines%23loc-laboratory-protocols/1/010f01942b67e5a5-12eb4139-1705-43ae-a2e9-24a9e5343e4b-000000/-Muma0DRWyqwJTQQcreaEwG6AgFeToNvmH6lpVo7o0Q=192). Additionally, PLOS ONE offers an option for publishing peer-reviewed Lab Protocol articles, which describe protocols hosted on protocols.io. Read more information on sharing protocols at [https://plos.org/protocols?utm_medium=editorial-email&utm_source=authorletters&utm_campaign=protocols](https://track.editorialmanager.com/CL0/https:%2F%2Fplos.org%2Fprotocols%3Futm_medium=editorial-email%26utm_source=authorletters%26utm_campaign=protocols/1/010f01942b67e5a5-12eb4139-1705-43ae-a2e9-24a9e5343e4b-000000/CkJGDpE0NE4ulqGpcw3Z47UW37fiLFyERX4AI5WA1og=192).

Response: The protocol was published. The title and doi are given below.

doi: 10.1186/s13063-021-05379-2.

**Title: Lowering blood pressure by changing lifestyle through a motivational education program: a cluster randomized controlled trial study protocol**

Journal Requirements:

1. When submitting your revision, we need you to address these additional requirements.

   Please ensure that your manuscript meets PLOS ONE's style requirements, including those for file naming. The PLOS ONE style templates can be found at
   [https://journals.plos.org/plosone/s/file?id=wjVg/PLOSOne_formatting_sample_main_body.pdf](https://track.editorialmanager.com/CL0/https:%2F%2Fjournals.plos.org%2Fplosone%2Fs%2Ffile%3Fid=wjVg%2FPLOSOne_formatting_sample_main_body.pdf/1/010f01942b67e5a5-12eb4139-1705-43ae-a2e9-24a9e5343e4b-000000/rbqQXo3GwLJUmAwCyP95riSrJV1hKluLTdr6CRDzFvA=192) and
   [https://journals.plos.org/plosone/s/file?id=ba62/PLOSOne_formatting_sample_title_authors_affiliations.pdf](https://track.editorialmanager.com/CL0/https:%2F%2Fjournals.plos.org%2Fplosone%2Fs%2Ffile%3Fid=ba62%2FPLOSOne_formatting_sample_title_authors_affiliations.pdf/1/010f01942b67e5a5-12eb4139-1705-43ae-a2e9-24a9e5343e4b-000000/X0NREgAC9LaFx6EQTEQ4UC4e7GspvDzOkvlybTTwkB0=192)
   Response: I have formatted according to the Journal’s requirements.

2. Please ensure you have included the registration number for the clinical trial referenced in the manuscript.

Response: **Trial registration:** ClinicalTrials.gov [NCT04505150](http://clinicaltrials.gov/show/NCT04505150) . Registered on 7 August 2020.

2. When completing the data availability statement of the submission form, you indicated that you will make your data available on acceptance. We strongly recommend all authors decide on a data sharing plan before acceptance, as the process can be lengthy and hold up publication timelines. Please note that, though access restrictions are acceptable now, your entire data will need to be made freely accessible if your manuscript is accepted for publication. This policy applies to all data except where public deposition would breach compliance with the protocol approved by your research ethics board. If you are unable to adhere to our open data policy, please kindly revise your statement to explain your reasoning and we will seek the editor's input on an exemption. Please be assured that, once you have provided your new statement, the assessment of your exemption will not hold up the peer review process.

Response: Data are now made available.

Data S1 HLS EU_PLOS One.xlsx
