# Supplementary material for: Study of the psychometric properties of the HLS-EU-12 questionnaire in rural Bangladesh
Source: PLoS One. 2025 May 12;20(5):e0323608. doi: 10.1371/journal.pone.0323608 (PMC12068572; doi:10.1371/journal.pone.0323608)
Supplement: Attachment — Submitted filename: Response to reviewers R2.docx [file pone.0323608.s007.docx]

PONE-D-24-39234R2

Study of the psychometric properties of the HLS-EU-12 questionnaire in rural Bangladesh.

PLOS ONE

Dear Professor Karl Bang Christensen,
Academic Editor, PLOS ONE

Thank you for your positive feedback on my manuscript.  Please see the responses below. Thank you for considering the manuscript very favorably for publication in PLOS One.

Your sincerely

Amirul Islam

**Comments to the Author**

4. Have the authors made all data underlying the findings in their manuscript fully available?

The [PLOS Data policy](http://track.editorialmanager.com/CL0/http:%2F%2Fwww.plosone.org%2Fstatic%2Fpolicies.action%23sharing/1/010f0195f89ecc35-39190cff-8152-488f-bf64-333c4506094b-000000/FiXIp1qVw-11CQ5E0UazGzcVoGP_P0R22RHCtqaHVzo=205) requires authors to make all data underlying the findings described in their manuscript fully available without restriction, with rare exception (please refer to the Data Availability Statement in the manuscript PDF file). The data should be provided as part of the manuscript or its supporting information, or deposited to a public repository. For example, in addition to summary statistics, the data points behind means, medians and variance measures should be available. If there are restrictions on publicly sharing data—e.g. participant privacy or use of data from a third party—those must be specified.

Reviewer #1: No

Reviewer #1: Dear author,
the revision improved the article, still the english, especially use of specific terms in the method and result section need to be more precise and unambiguous.
Not all my comments have been addressed, especially my point on local item dependencies was ignored. Based on the table with residual correlations, there are no local item dependencies in this scale (cut-off 0.3). For the local item dependencies the correlations have to be bigger than the cut-off. The authors chose 0.3, but are there correlations > 0.3. If I believe the table, there are correlations < -0.3 (of - 0.33) but negative correlations are not of interest when analysing the local item dependencies.

Response: Thanks. I have included the following section in the method section. I have made this correction in the discussion section.

Local Independence

The local independence (LD) can be tested by calculating the correlation coefficients among residuals, which are expected to be unrelated and generally close to zero [54]. Violations of LD can lead to inflated estimates of reliability and problems with construct validity. Currently, no well-documented suggestions of a single critical value indicate LD. Marais and Andrich[55] investigated dependence at a critical value of 0.1, but a value of 0.3 has often been used [56-58]; critical values of 0.5 [59, 60] and even 0.7 [61] were also found to be in use. Christensen et al. [62] conducted a comprehensive study to identify LD in the Rasch model using residual correlations and reported that LD depended on several factors, including the number of items, number of response categories, and the number of respondents. For example, for polytomous items, the critical value for LD was approximately 0.11 for 10 items, 0.18 for 15 items, and 0.21 for 20 items and 300 participants for each item condition. The study reported a minor effect on the number of items, with a decrease in critical values with an increase in sample size.

*******************************
I suggest to the author to re-read the article more carefully, to reflect on the phrasing of methods and results. Following points have retained my attention when reading the manuscript:
Response: Now the manuscript is updated.
General comment,
the statistical analyses per se are fine for me (except Local item dependency), however the language is not yet precise enough and the authors should invest some more time in writing everything down properly. Also, for each of the analysis done (Targeting, DIF, LID etc), the author could report the before and after deleting item 12.

|  | 12 items | 11 items |
| --- | --- | --- |
| Unidimensionality test (% that goes beyond 95% CI) | 5.2% CI (2.8 – 7.7) | 5.2% CI (2.8 – 7.8) |

Response: (i) The Unidmensionality was tested for 12 and 11 items as below (Table 2, last row)

(ii) The DIF analysis for 11 items did not change the significance levels for age and gender. The P values for gender only are shown in Table 3 (last column).

(iii) Local Dependency: Residual correlation coefficients for 12 items, lower triangular part, and 11 items, upper triangular part, are shown in Table 4.

(iv) Targeting for 11 items is also shown as below (Fig. 3)

Abstract: "categorization of items" – do you mean the analysis of the ordering of the response options?

"The analysis supported the scale’s unidimensionality as reported by the lower bound of a binomial 95% confidence interval (CI) of the observed proportion 5.2." – The phrasing is confusing, it sounds like 5.2 is the lower bound.

Response: Now it is written as (page 21):

The Unidimensionality of HLS-EU-12 was tested using principal component analysis (PCA) residuals (5.2%, 95% CI 2.8% to 7.7%) for total items and (5.2%, 95% CI 2.8% to 7.8%) for 11 items, excluding item 12 "..join a sports club or exercise class if you want to.." indicating the lower bound did not fall within the 5% of the critical region (Table 2 (last row)), which supports the Unidimensionality of the HLS-EU-12 item.

Materials and methods – the authors write that the analysis "was focused on invariance – including differential item functioning across gender and age." Why focused on invariance? and what other methods beside DIF are interested in invariance (- because of writing "including DIF").

Response: I have simplified it as:

The analysis was focused on differential item functioning (DIF) across gender and age, targeting, multidimensionality, response dependency, and item categorization.

Introduction


Please rewrite: In the study location, people have minimal knowledge of the most common chronic diseases, such as diabetes, common eye diseases, and mental health[29, 30] mental health [31].

Response: I have simplified this as

Previous studies have reported that rural people did not have sufficient knowledge of the most common chronic diseases, such as diabetes, common eye diseases, and mental health [29-31].

Unidimensionality:

Please rewrite: "Compare the two estimates on a person-by-person basis using t-tests to determine the number of cases that differ significantly at the 0.05 level or the lower bound of a binomial 95% confidence interval (CI) of the observed proportion overlaps 5%. It is suggested a unidimensionality [53]."

Response: To make it clearer, I have written it as

One of the assumptions of Rasch analysis is unidimensionality, which occurs when all items measure only one common attribute only [52]. Three steps are involved in order to test unidimensionality within the Rasch model:

1. Identify two item sets from a Principal Component Analysis (PCA) of residuals

2. Estimate separate person measures based on the two item sets

3. Compare the two estimates on a person-by-person basis using *t*-tests and determine the number of cases that differ significantly at the 0.05-level.

If ≤5% of tests are significant, or the lower bound of a binomial 95% confidence interval (CI) of the observed proportion overlaps 5%, then the scale is considered unidimensional. Otherwise, the scale is regarded as multidimensional [53].

Local Independence:
The local independence can be tested by calculating the correlation coefficients among item residuals, which are expected to be unrelated / independent / free of any associations [54].

Response: I have written this as

The local independence (LD) can be tested by calculating the correlation coefficients among residuals, which are expected to be unrelated and generally close to zero [54]. Violations of LD can lead to inflated estimates of reliability and problems with construct validity. Currently, no well-documented suggestions of a single critical value indicate LD. Marais and Andrich[55] investigated dependence at a critical value of 0.1, but a value of 0.3 has often been used [56-58]; critical values of 0.5 [59, 60] and even 0.7 [61] were also found to be in use. Christensen et al. [62] conducted a comprehensive study to identify LD in the Rasch model using residual correlations and reported that LD depended on several factors, including the number of items, number of response categories, and the number of respondents. For example, for polytomous items, the critical value for LD was approximately 0.11 for 10 items, 0.18 for 15 items, and 0.21 for 20 items and 300 participants for each item condition. The study reported a minor effect on the number of items, with a decrease in critical values with an increase in sample size.

Differential Item Functioning:

"to test the Differential Item Functioning (DIF) of the tool." -> to test the absence of "Differential Item Functioning in the tool. The abbreviation does not need to be repeated.
Response: I have used the abbreviation after the first spell out.

Table 2 – why not write the footnote to this table directly into the table?

Response: It is done (Table 2)

Results:
Unidimensionality – this section mixes methods and results. What happens with the dimensionality after deleting item 12?

Response: This section is written as:

The Unidimensionality of HLS-EU-12 was tested using principal component analysis (PCA) residuals for total items (5.2%, 95% CI 2.8% to 7.7%) and 11 items, excluding item 12 "..join a sports club or exercise class if you want to.." (5.2%, 95% CI 2.8% to 7.8%) indicating the lower bound did not fall within the 5% of the critical region (Table 2 (last row)), which supports the Unidimensionality of the HLS-EU-12 item.

Local Dependency:
What do the authors mean with regard to the creation of the subtests that it "did not improve the overall model". It can be expected that the PSI goes down, because the dependent items inflate the reliability. In that sense, I would recommend making the subtests if there are correlations > 0.3.

Response: None of residual correlation coefficients was greater than 0.13 except for one was 0.20. Therefore, no subset analyses were conducted.

However, with regard to table 4, I am wondering if the authors are also considering negative correlations. For the LID only high positive correlations are relevant. In that sense, if I am reading Table 4 correctly, the highest positive correlation is between item HLQ5 and HQL9 with r = 0.2 ? – I would rather discuss this dependency. The cut-off of 0.3 is very high.

Response: I have discussed the correlation between item HLQ5 and HQL9 with r = 0.2 as per Christensen et al. (2017).

## Targeting could the authors also add what it means, for example, if there is a higher frequency of female participants towards the left, that they have lower health literacy ? (or reverse?) Response: Now it is written as: The figures show that the item set, and the persons are well spread around the mean, with a higher frequency of female distribution towards the left, indicating that the mean person location is less than zero or below average level of health literacy (-0.337 for total items and -0.336 for 11 items) and a higher frequency of male distribution towards the right, indicating that the mean person location is more than zero or above average level of health literacy (0.175 for total items and 0.204 for 11 items). However, the wide spread of person and item locations suggests that the participants are well-targeted to items.

Fig 2. Typo – Perosn-item -> Person-item

Response: Thanks. It is corrected.

Discussion-

Please correct – to what I read in the table, they are no correlations greater than 0.3. Also item 12's fit residual was significant = item 12 was misfitting.

"sufficient psychometric properties" -> had good psychometric properties?

Response: It is corrected.

7. PLOS authors have the option to publish the peer review history of their article ([what does this mean?](https://track.editorialmanager.com/CL0/https:%2F%2Fjournals.plos.org%2Fplosone%2Fs%2Feditorial-and-peer-review-process%23loc-peer-review-history/1/010f0195f89ecc35-39190cff-8152-488f-bf64-333c4506094b-000000/Y3sFlDLSr2wiatbH7FrsQQ2k0iQkIZkD4e-MJucFPbk=205)). If published, this will include your full peer review and any attached files.


If you choose “no”, your identity will remain anonymous but your review may still be made public.


**Do you want your identity to be public for this peer review?** For information about this choice, including consent withdrawal, please see our [Privacy Policy](https://track.editorialmanager.com/CL0/https:%2F%2Fwww.plos.org%2Fprivacy-policy/1/010f0195f89ecc35-39190cff-8152-488f-bf64-333c4506094b-000000/YdPLXzn0p_d8PXKtpLTT0wfORzCEVYGfuKPDt9lu3Rw=205).

Reviewer #1: No
